# Identification and artificial cultivation of a wild strain of *Agaricus sinodeliciosus*

**DOI:** 10.1515/biol-2025-1365

**Published:** 2026-08-18

**Authors:** Yanyan Xu, Shasha Feng, Jinping Lan, Longzuo Xin

**Affiliations:** College of Agriculture and Forestry Technology, Hebei North University, Zhangjiakou, Hebei 075131, China; Life Science Research Center, Hebei North University, Zhangjiakou, Hebei 075032, China

**Keywords:** *Agaricus sinodeliciosus*, identification, cultivation, domestication

## Abstract

A wild strain collected from the Xinjiang Uygur Autonomous Region underwent morphological observation and molecular biological identification, followed by an investigation into its biological and cultural characteristics. The results identified the strain as *Agaricus sinodeliciosus*, with an optimal nitrogen source of cow manure at 10 g/L and an optimal carbon-to-nitrogen (C/N) ratio of 30:1. The suitable temperature range for mycelial growth was 21–27 °C, with an optimal pH value of 7. The recommended stock culture medium comprised 53.86 % wheat, 1.74 % lime, 4.4 % cow manure, and 40 % straw fermented material, with a pH of 7.5. The optimal medium for cultivation consisted of 30 % corn straw, 23.5 % corncob, 45 % cow manure, and 1.5 % lime, with a C/N ratio of 30:1, a pH of 7.5, and 65 % moisture content. *A. sinodeliciosus* can fruit through three cultivation methods: soilless, soil-covered, and frame-based cultivation. This study explores and optimizes the biological characteristics and cultivation conditions of *A. sinodeliciosus*, providing a reference for its large-scale production.

## 1 Introduction

*Agaricus sinodeliciosus* is a rare large edible fungus native to northwest China [1]. The wild fruiting bodies of this species grow in soil, emitting a strong aroma and possessing a delicate flavor, offering both edible and medicinal value [1]. The protein content of *A. sinodeliciosus* fruiting bodies can reach 40.86 %, with 20.68 % dietary fiber, and they are rich in vitamins B and D [2], 3]. Furthermore, petroleum ether extracts from *A. sinodeliciosus* exhibit *in vitro* antitumor activity [4]. Extracellular polysaccharides (EPS) from *A. sinodeliciosus* can mitigate neuroinflammation, oxidative damage, and cognitive impairment in AD rats by reshaping their gut microbiota [5], 6].

Due to *A. sinodeliciosus*’s high market value and nutritional benefits, residents have engaged in excessive collection of wild *A. sinodeliciosus*, causing severe damage to its native ecological environment [7]. In response, the Ebinur Lake Wetland, one of the key habitats for *A. sinodeliciosus*, was designated as an autonomous regional-level nature reserve in June 2000 and later upgraded to a national-level nature reserve in April 2007. Although *A. sinodeliciosus* is not the primary protection target, the establishment of the reserve indirectly safeguards its survival by protecting the ecological environment [8].

In recent years, the domestication and cultivation of *A. sinodeliciosus* have become a focal point in edible fungus research. Many wild populations of *A. sinodeliciosus* are now difficult to find in their native habitats, and their rich genetic resources face the risk of continuous loss. Additionally, the wild fruiting bodies of *A. sinodeliciosus* often grow underground, appearing dirty and morphologically diverse, which affects their market appeal. Their long reproductive cycle further limits their commercial potential. Currently, *A. sinodeliciosus* has been successfully domesticated, enabling soilless [9], film-bag [10], and covered soil cultivation [11]. Further exploration of cultivation methods and management practices is necessary to enhance the yield and quality of *A. sinodeliciosus*.

This research team collected a wild strain of *A. sinodeliciosus* from the Xinjiang Uygur Autonomous Region and conducted systematic identification through morphological observation and molecular biology methods. To optimize cultivation conditions, we determined the optimal nitrogen source and C/N ratio for mycelial growth and explored the effects of different cultivation methods on yield and agronomic traits. The aim is to meet market demand for new edible fungi through artificial domestication while effectively protecting wild resources. The research findings hold significant importance for advancing the commercialization of *A. sinodeliciosus*, enriching the edible fungus market supply, and achieving a dual win in ecology and economy.

## 2 Materials and methods

### 2.1 Strain isolation, cultivation, and morphological observation

*Agaricus sinodeliciosus* MW01 (CGMCC 5.2277) was isolated from a wild specimen collected from Xinyuan County, Ili Kazakh Autonomous Prefecture, Xinjiang Uygur Autonomous Region, China. Strains were stored in the Edible Fungus Laboratory of Hebei North University. The strains were kept at 4 °C and cultivated on potato dextrose agar (PDA) at 25 °C in the dark. The basidiospore and mycelium were observed by hemocytometer under a Nikon Eclipse 80i microscope (Nikon, Tokyo, Japan).

### 2.2 The identification of the strains

DNA was extracted from the mycelia using the CTAB method [12]. The fungal rDNA internal transcribed spacer (ITS) region was amplified by PCR using universal primers ITS1 (5′-TCG​TAG​GTG​AAC​CTG​CGG-3′) and ITS4 (5′-TCT​CCC​GCT​TAT​TGA​TAT​GC-3′). A 20 μL PCR reaction system was used, with the annealing temperature of primers set at 55 °C. The amplified sequences were verified by agarose gel electrophoresis, then submitted to Tianyi Huiyuan Biotechnology Co., Ltd. for sequencing.

The ITS sequences were submitted to the GenBank database (accession numbers PV496989) and subjected to blastn analysis within the NCBI platform. Top-hit sequences (E-value < 1e-50) with ≥ 97 % identity were retrieved for phylogenetic reconstruction. These alignments were manually refined and end-trimmed to remove poorly aligned and divergent regions. Unambiguously aligned positions were then utilized for constructing phylogenetic trees with maximum likelihood (ML) methods. The optimal evolution model was determined by Modelfinder as GTR + G for ML analysis. An ML tree was constructed using MEGA X (gap treatment: use all sites; 1,000 bootstrap replications) [13].

### 2.3 Determination of optimal temperature and pH

Activated mycelial disks (5 mm × 5 mm) were inoculated onto PDA plates and incubated at 18, 21, 24, 27, 30, and 35 °C in the dark, with three replicates per treatment. Mycelial diameter was measured using the cross-over method. Similarly, activated mycelial disks were inoculated onto PDA plates adjusted to pH 5, 6, 7, 8, and 9, with PDA plates as the control (CK), and incubated at 25 °C in the dark, with three replicates per treatment. Mycelial diameter was measured using the cross-over method.

### 2.4 Determination of optimal nitrogen source

Using potato dextrose agar (PDA) as the basal medium, five nitrogen sources were added respectively: yeast powder, peptone, carbon-enzyme protein organic fertilizer (Kuretada Biotechnology Co., Ltd.), cow manure, and beef extract. The supplementation levels for yeast powder, peptone, and beef extract were set at 5, 10, and 15 g, while for carbon-enzyme protein organic fertilizer, the levels were 1, 3, and 5 g. The control group (CK) consisted of PDA plates without any added nitrogen source. Each treatment was replicated three times. Mycelial growth was periodically monitored, and the diameter of the mycelium was measured using the cross-over method [14].

### 2.5 Determination of optimal carbon-to-nitrogen ratio

Basal medium: 20 g/L of glucose, 18 g/L of agar, and 1,000 ml of distilled water. Add peptone in proportion to adjust the carbon-to-nitrogen ratio. The carbon-to-nitrogen ratios of the test media are set to 10:1, 15:1, 20:1, 25:1, 30:1, 35:1, 40:1, 45:1, and 50:1 respectively. Each treatment was replicated three times.

Calculation formula:X=TC/E

*X* – amount of peptone added (g); TC – total carbon content (20 g); *E* – target C/N ratio.

The glucose used in the experiment is analytically pure glucose with a C content of 100 %, and the N content of the peptone (Shanghai Macklin Biochemical Co., Ltd., CAS: 68308-36-1) used is 12 %. Based on 20 g of glucose, the corresponding peptone addition amounts are 16.76 g/L, 11.08 g/L, 8.33 g/L, 6.67 g/L, 5.56 g/L, 4.76 g/L, 4.17 g/L, 3.70 g/L, and 3.33 g/L. The medium without additional peptone is used as the control group (CK) [14].

### 2.6 Determination of lignin-degrading enzyme activity

The mycelial discs (8 mm × 8 mm) were inoculated onto PDA–guaiacol medium, PDA–aniline blue medium, PDA–manganese chloride medium, and PDA plates (CK), respectively, using the plate inoculation method. Each treatment was replicated three times. After incubation at 25 °C for 9 days, the presence of reddish-brown oxidative rings, decolorization zones, and brownish-brown coloration rings was observed, and their diameters were recorded. This was performed to verify the ability of *A. sinodeliciosus* MW01 to produce laccase (Lac), lignin peroxidase (Lip), and manganese peroxidase (Mnp) [15]. The following are the three types of culture media needed. PDA–aniline blue medium (I): 200 g peeled potatoes, 20 g glucose, 0.4 g aniline blue, 3.0 g KH_2_PO_4_, 1.5 g MgSO_4_, 18 g agar, 1,000 mL distilled water, pH 7.5–8.0; PDA–manganese chloride medium (II): 200 g peeled potatoes, 20 g glucose, 0.15 g MnCl_2_·4H_2_O, 3.0 g KH_2_PO_4_, 1.5 g MgSO_4_, 18 g agar, 1,000 mL distilled water, pH 7.5–8.0; PDA–guaiacol medium (III): 200 g peeled potatoes, 20 g glucose, 0.4 mL guaiacol, 3.0 g KH_2_PO_4_, 1.5 g MgSO_4_, 18 g agar, 1,000 mL distilled water, pH 7.5–8.0.

### 2.7 Determination of suitable stock culture medium formulas

The seven formula ingredients were thoroughly mixed in the specified proportions. The overall moisture content was controlled at 60 %, and the mixture was then packed into 200 mL spawn bottles. These bottles were sterilized in an autoclave at 121 °C for 2 h. After cooling, the spawn bottles were transferred to a laminar flow hood, allowed to cool to room temperature, and then subjected to UV sterilization for 15 min before inoculation [10]. Equal amounts of mycelial blocks were inoculated into culture bottles containing seven different substrate formulations, respectively. The spawn bottles were then placed in a spawn cultivation chamber (maintained at 22 °C) and cultured in the dark. Periodic observations were conducted to monitor mycelial growth vigor and measure mycelial length. The following are the seven formulations of stock culture media to be comparatively evaluated. Formula I: Wheat 53.86 %, lime 1.74 %, cow manure 4.4 %, fermented substrate 40 %, pH 7.5. Formula II: Cottonseed hulls 61 %, wheat bran 9.8 %, cow manure 26 %, gypsum 0.8 %, lime 0.8 %, light calcium carbonate 0.8 %, superphosphate 0.8 %, pH 7.5. Formula III: Corn cob 49 %, wheat bran 49 %, lime 1 %, gypsum 1 %, pH 7.5. Formula IV: Fermented substrate 77 % (C/N = 30:1), wheat grains 30 %, lime 1 %, gypsum 1 %, pH 7.5. Formula V: Fermented substrate 98 % (C/N = 30:1), lime 1 %, gypsum 1 %, pH 7.5. Formula VI: Fermented substrate 77 % (C/N = 45:1), wheat grains 30 %, lime 1 %, gypsum 1 %, pH 7.5. Formula VII: Fermented substrate 98 % (C/N = 45:1), lime 1 %, gypsum 1 %, pH 7.5.

### 2.8 Bottle cultivation

After the spawn bottles were fully colonized, they were transferred to the cultivation chamber for weak light stimulation to induce primordium formation. Two treatment protocols were implemented. Non-soil cover treatment: Following primordium emergence, the temperature was adjusted to 18–22 °C. When the primordia had reached approximately 3 cm in height, the bottle caps were removed to expose them to ambient conditions. Air humidity was maintained at 80%–90 %, with ventilation performed 3 times daily (30 min per session). Soil cover treatment: After mycelium had reached the soil surface layer, the cultivation chamber temperature was controlled at 18–22 °C, with the same ventilation regimen (3 × 30 min/day) to stimulate primordium formation. Ten to fifteen days later, first flush harvesting was conducted according to *Agaricus bisporus* harvesting standards: fruiting bodies were collected before pileus opening, with a pileus diameter of approximately 50 mm. After harvesting, the culture was transitioned to a second flush cultivation phase. Throughout the process, the growth conditions of cultivated fruiting bodies were monitored, and agronomic traits (e.g., pileus diameter, stipe length) were measured [9].

### 2.9 Covered soil frame cultivation

Frame cultivation medium: 30 % corn straw, 23.5 % corncob, 45 % cow manure, 1.5 % lime, pH 7.5, C/N ratio adjusted to 30:1, moisture content 65 %. Aerobic fermentation of the cultivation substrate was carried out using a laboratory-scale negative-pressure oxygen-enriched fermentation system, with the substrate moisture content maintained at approximately 65 %. The fermented material was loaded into white cultivation trays to half their height, cooled to room temperature, and then inoculated with spawn. The cultivation chamber was maintained at 18–22 °C with 65–75 % relative humidity. After covering with plastic film, the cultures were incubated in darkness. Mycelial germination occurred within approximately 2 days, and by day 23, the mycelium had fully colonized the substrate. A 4 cm soil layer was then applied, and within 4–6 days, the mycelium had reached the soil surface. When humidity was adjusted to 80–95 %, primordia began to form and develop after 12 days. Harvesting of the first flush was conducted when the fruiting body pileus reached approximately 50 mm in diameter. The cultivation cycle was repeated until fruiting ceased. Throughout the process, developmental progress and agronomic traits (including pileus diameter, stipe length, and fresh weight) of the cultivated fruiting bodies were systematically observed and measured [9].

## 3 Results

### 3.1 Strain MW01 microscopic morphological characteristics

**Figure 1:**
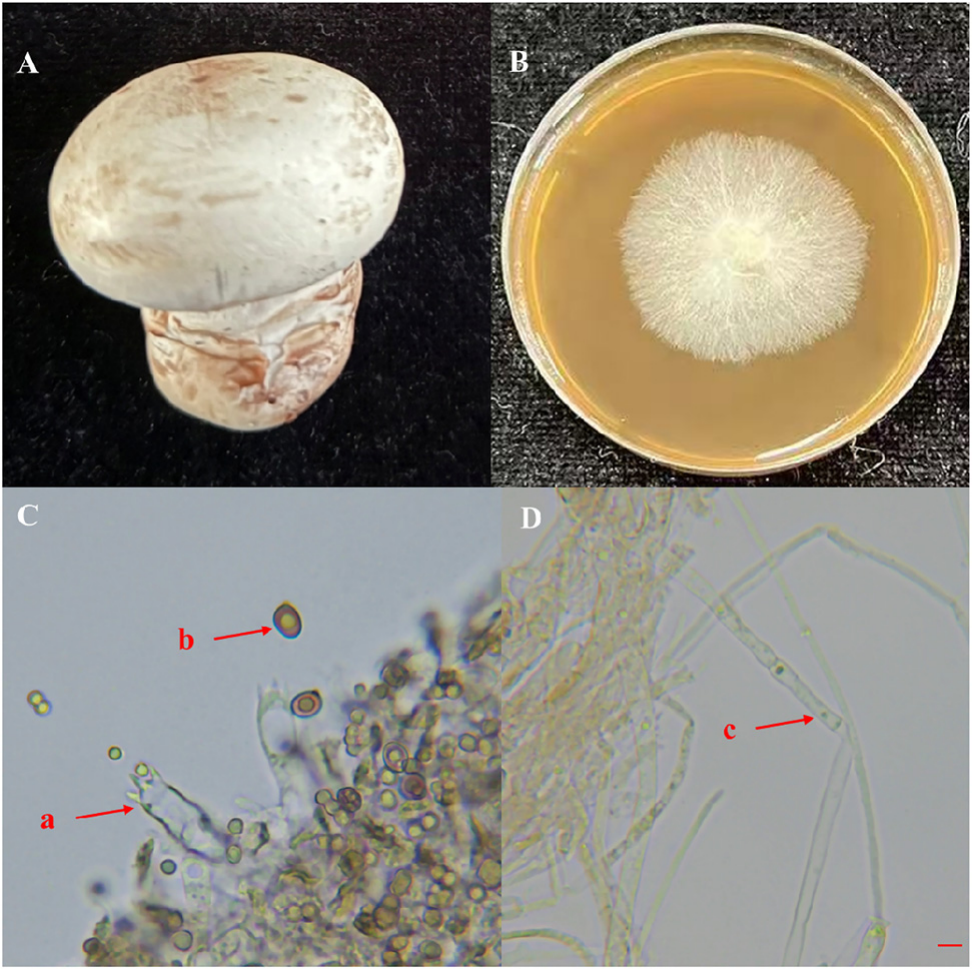
Morphological characteristics of fruiting bodies and mycelium of *Agaricus sinodeliciosus MW01* (bar = 10 μm). A: fruit body; B: colony morphology; C: lamella; D: hyphal micromorphology; a: basidium; b: basidiospore; c: no clamp connection.

The pileus (cap) of the fruiting body is nearly white in color, exhibiting a flattened hemispherical shape. The central region of the pileus is covered with a layer of light brown scales, while the pileus margin demonstrates an inward-rolling state (Figure 1A). The context (flesh) is white with a relatively dense texture. The stipe (stem) originates from the central pileus position and appears robust. The gills are initially white, gradually transitioning to pinkish hues before ultimately developing into dark brown, with unequal gill lengths. Additionally, a ring is present at the midpoint of the stipe. Mature basidiospores are brown in color, featuring a smooth surface and an elliptical to ovoid shape, measuring (6.0–7.5) μm × (5.5–6.5) μm (Figure 1C). Through tissue isolation and pure culture of this fruiting body, the resulting colony exhibits a regular circular shape with a slightly convex center. The mycelium appears pure white and gradually flattens over time (Figure 1B). Microscopic examination reveals the absence of prominent clamp connections in the hyphae (Figure 1D).

### 3.2 Strain MW01 was identified as *A. sinodeliciosus* by phylogenetic analysis based on the ITS sequences

**Figure 2:**
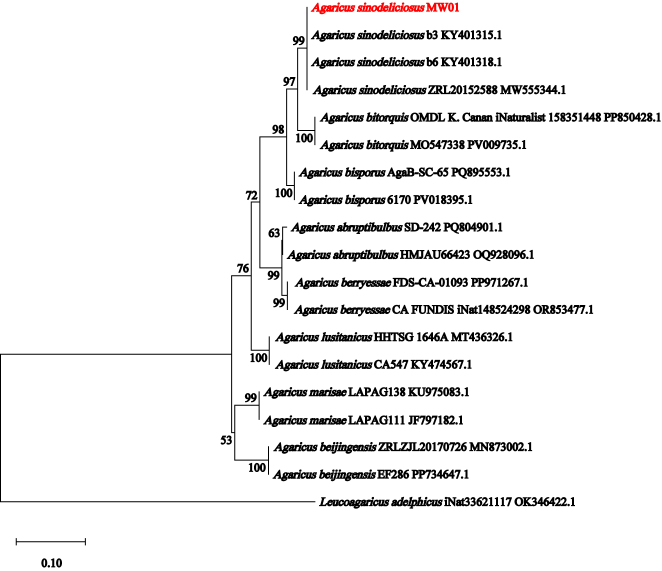
Phylogenetic tree of *Agaricus sinodeliciosus* MW01.

The ITS sequence of strain MW01 was obtained and submitted to GenBank under the accession number PV496989. A total of 19 ITS sequences were used to construct phylogenetic trees. Modelfinder identified GTR + G as the best fit model for ML analysis. Phylogeny analysis based on the ITS sequences confirmed that strain MW01 belongs to *A. sinodeliciosus* (Figure 2). The ITS sequence of *Leucoagaricus adelphicus* iNat33621117 (OK346422.1) was used as the outgroup.

### 3.3 Biological characteristics

The *A. sinodeliciosus* MW01 strain exhibits optimal growth at 27 °C, with robust mycelial development observed within the temperature range of 21–27 °C. Although mycelial growth persists at 35 °C, the growth rate decreases significantly under this condition. When cultured at 25 °C, the strain demonstrates adaptability to media with pH values ranging from 5 to 9, with the fastest growth rate (1.43 mm/day) observed at pH 7 (Table 1).

**Table 1:** Biological characteristics of *Agaricus sinodeliciosus* MW01.

Temperature	Growth rate (mm/d)	pH	Growth rate (mm/d)
18 °C	0.590 ± 0.00 d	5	0.75 ± 0.04 d
21 °C	1.2 ± 0.07 c	6	1.22 ± 0.02 b
24 °C	1.32 ± 0.07 bc	7	1.43 ± 0.08 a
27 °C	1.63 ± 0.02 a	8	0.92 ± 0.07 c
30 °C	0.57 ± 0.03 d	9	0.81 ± 0.03 cd
35 °C	0.25 ± 0.02 e	CK	1.36 ± 0.03 a

The same lowercase letters in the same column represent no significant difference (*p* < 0.05).

### 3.4 Effects of different nitrogen sources on mycelial growth of strain MW01

When cow manure was used as the nitrogen source, *A. sinodeliciosus* MW01 exhibited the fastest mycelial growth rate and most vigorous development, followed by peptone and yeast extract. The slowest growth occurred on PDA medium (without supplemental nitrogen). Optimal mycelial growth was observed with 5 g/L concentrations of yeast extract, peptone, and beef extract, all of which significantly outperformed higher nitrogen concentrations. Consequently, the optimal nitrogen source for *A. sinodeliciosus* MW01 was determined to be 10 g/L cow manure (Table 2).

**Table 2:** Effects of different nitrogen sources on mycelial growth rate and growth potential of *Agaricus sinodeliciosus* MW01.

Nitrogen source	Proportion (g/L)	Growth rate (mm/d)	Mycelium growth condition
PDA	0	0.85 ± 0.05 i	++
PDA + yeast extract	5	1.40 ± 0.05 abcd	+++
	10	1.32 ± 0.05 de	++
	15	1.19 ± 0.05 g	++
PDA + peptone	5	1.34 ± 0.06 cde	+++
	10	1.37 ± 0.05 bcd	++
	15	1.28 ± 0.05 ef	+
PDA + beef extract	5	1.43 ± 0.06 ab	+++
	10	1.38 ± 0.05 abcd	++
	15	1.22 ± 0.06 fg	++
PDA + cow manure	5	1.42 ± 0.07 abc	+++
	10	1.46 ± 0.06 a	+++
	15	1.24 ± 0.07 fg	+++
PDA + carbon-enzyme	1	1.38 ± 0.03 abcd	++
Protein organic fertilizer	3	1.41 ± 0.06 abc	+++
	5	1.04 ± 0.08 h	++

The same lowercase letters in the same column represent no significant difference (*p* < 0.05).

### 3.5 Determination of optimal carbon-to-nitrogen (C/N) ratio for mycelial growth of strain MW01

Within the tested C/N ratio range of 10:1 to 50:1, *A. sinodeliciosus* MW01 demonstrated germination within 48 h post-inoculation. After complete plate colonization, the mycelium exhibited a velvety, white, circular morphology. Significant differences in growth rates were observed across varying C/N ratios. Optimal growth performance was achieved at C/N ratios of 35:1, 30:1, and 25:1, characterized by dense and robust mycelial development. Other C/N ratio treatments showed comparable growth patterns, all failing to reach the performance levels observed under the three aforementioned ratios. Under nitrogen-free conditions (CK) using glucose as the carbon source and agar medium as the substrate, mycelial growth was sparse with the slowest growth rate recorded at 0.56 mm/day (Table 3). These findings indicate that when glucose serves as the carbon source and peptone as the nitrogen source, *A. sinodeliciosus* MW01 exhibits relatively flexible C/N ratio requirements, maintaining satisfactory growth across the tested range. However, mycelial vigor demonstrated significant variation depending on the C/N ratio, with the densest and most vigorous growth observed at 35:1, 30:1, and 25:1 ratios.

**Table 3:** Determination of the optimal carbon nitrogen ratio for the growth of *Agaricus sinodeliciosus* MW01.

Treatment	Growth rate (mm/d)	Mycelium growth condition
C/N 10:1	0.77 ± 0.01 a	++
C/N 15:1	0.77 ± 0.02 a	++
C/N 20:1	0.76 ± 0.01 a	++
C/N 25:1	0.78 ± 0.01 a	+++
C/N 30:1	0.78 ± 0.01 a	+++
C/N 35:1	0.79 ± 0.02 a	+++
C/N 40:1	0.77 ± 0.01 a	++
C/N 45:1	0.78 ± 0.02 a	++
C/N 50:1	0.77 ± 0.01 a	++
CK	0.56 ± 0.02 b	+

The same lowercase letters in the same column represent no significant difference (*p* < 0.05).

### 3.6 Lignin-degrading enzyme activities of strain MW01

**Figure 3:**
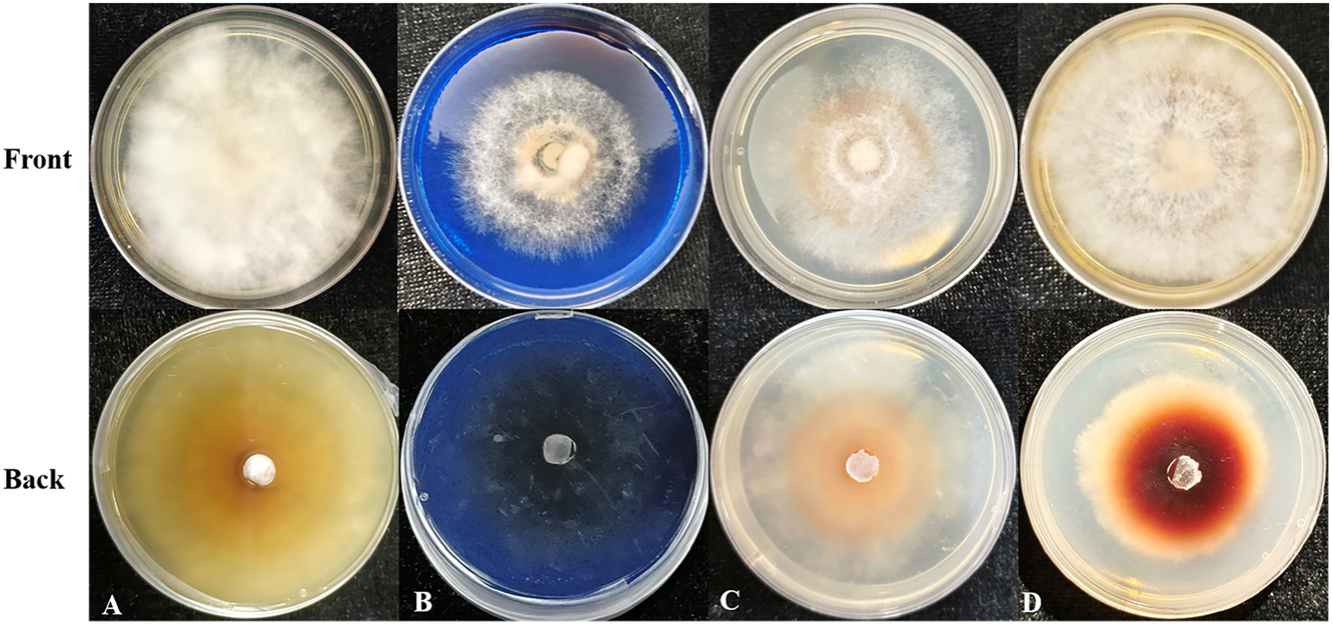
Growth of *Agaricus sinodeliciosus* MW01 on different media. A: PDA medium; B: PDA–aniline blue medium (I); C: PDA–manganese chloride medium (II); D: PDA–guaiacol medium (III).

On PDA–aniline blue medium (I) plates, partial decolorization occurred in the interaction zones of *A. sinodeliciosus* MW01 (Figure 3B), indicating limited secretory capacity for lignin peroxidase (Lip). When inoculated on PDA–manganese chloride medium (II) plates, the strain produced distinct pinkish coloration zones (Figure 3C), confirming its ability to secrete manganese peroxidase (Mnp). On PDA–guaiacol medium (III), the strain formed prominent orange-red coloration zones with relatively large diameters (Figure 3D), demonstrating strong laccase (Lac) production capacity and high secretory activity.

### 3.7 Determination of suitable stock culture medium formulas of strain MW01

*Agaricus sinodeliciosus* MW01 exhibited the fastest growth rate (1.47 mm/d) in original spawn Formula I (wheat + fermented substrate), with dense, robust, and pure white mycelial development (Table 4). In contrast, slower growth rates and sparser mycelium were observed in Formulas II and III. The optimized Formula IV (C/N = 30:1), achieved through strict C/N ratio control, reduced wheat grain content, and increased straw fermentation substrate proportion, demonstrated superior performance. In Formula IV, the mycelium grew so vigorously that primordia emerged when the culture bags reached half-full capacity. Formula V (0 % wheat grain), despite maintaining an optimal C/N ratio and producing dense white mycelium, showed significantly reduced growth rates (Table 4). Formula VII (C/N = 45:1), which also excluded wheat grains, exhibited rapid growth but produced sparse mycelia. Therefore, Formula IV (C/N ratio = 30:1) is deemed optimal as the stock culture medium for industrial production.

**Table 4:** Comparison of growth state of *Agaricus sinodeliciosus* MW01 under different stock culture formulas.

Formula	Growth rate (mm/d)	Mycelium growth condition
I	1.47 ± 0.08 a	White, thick, and vigorous
II	0.56 ± 0.06 b	White, sparse, and grows sluggishly
III	0.62 ± 0.04 b	Yellowish, sparse, and grows sluggishly
IV	1.67 ± 0.08 a	White, thick, and vigorous
V	0.76 ± 0.06 b	White and thick but grows sluggishly
VI	0.77 ± 0.08 a	White and thick but grows slowly
VII	1.47 ± 0.04 b	Sparse and grows sluggishly

The same lowercase letters in the same column represent no significant difference (*p* < 0.05).

### 3.8 Determination of optimal cultivation methods for strain MW01

**Figure 4:**
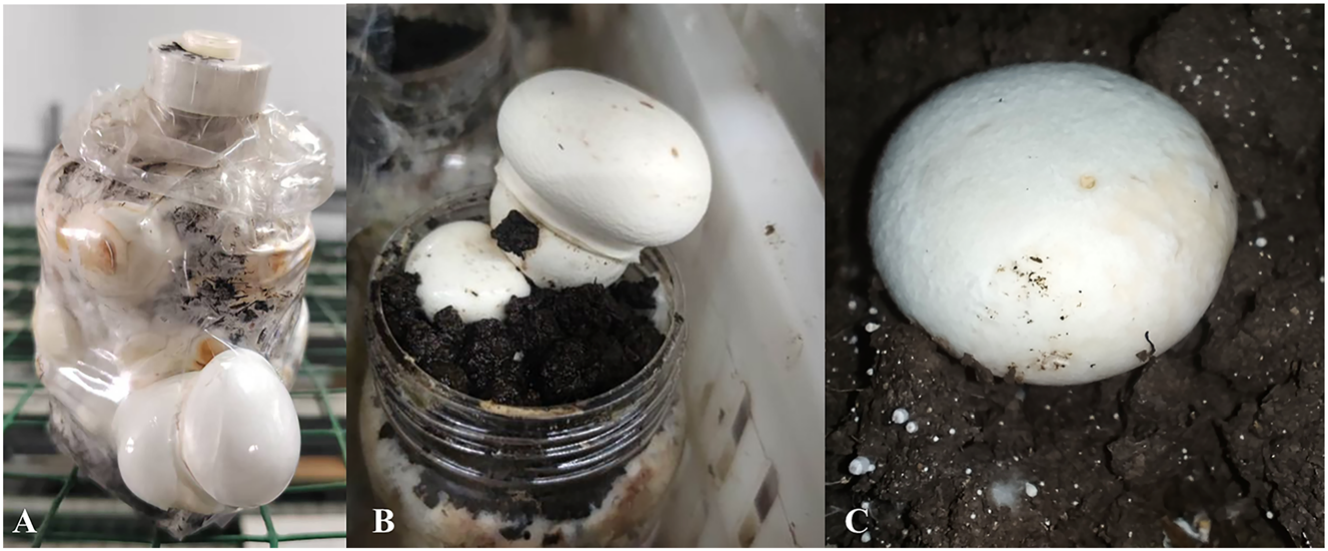
Three cultivation methods of *Agaricus sinodeliciosus*. A: soilless cultivation; B: soil covered bottle cultivation; C: frame mulching cultivation.

Three cultivation methods – soilless cultivation, covered-soil bottle cultivation, and frame cultivation – were evaluated in fruiting experiments. All methods successfully induced two fruiting flushes (Figure 4). In the case of the original spawn Formula IV of *A. sinodeliciosus*, primordia emerged before the bags were fully colonized by the mycelium. The bags were opened immediately after being fully colonized to induce fruiting. The temperature was maintained at 18–22 °C, the humidity at 90–95 %, and ventilation was carried out three times a day.

For covered-soil bottle cultivation, a 2–4 cm soil layer was applied, with a total growth cycle of approximately 70 days. Each bottle produced two fruiting flushes, with harvests conducted according to *Agaricus bisporus* standards (closed pileus, 5 cm diameter, ∼30 g/mushroom; Table 5). Biological efficiency reached 30.7 % in soilless bottle cultivation and 39.7 % in covered-soil bottle cultivation.

**Table 5:** Determination of agronomic traits of fruit body of *Agaricus sinodeliciosus* MW01 under different cultivation methods.

Treatment	Stipe length/cm	Stipe diameter/cm	Pileus diameter/cm	Pileus thickness/cm	Single mushroom weight/g
Soilless cultivation	2.40 ± 0.04 a	2.85 ± 0.03 a	4.95 ± 0.13 a	2.10 ± 0.04 a	20.95 ± 2.4 b
Covered-soil bottle cultivation	2.41 ± 0.02 a	2.92 ± 0.04 a	5.23 ± 0.12 a	2.18 ± 0.05 a	33.70 ± 3.7 a
Frame cultivation	2.49 ± 0.03 a	2.68 ± 0.03 b	5.21 ± 0.08 a	2.11 ± 0.02 a	29.70 ± 2.4 a

As mentioned above.

In frame cultivation, colonized spawn blocks were transferred to *Agaricus bisporus* cultivation chambers (18–22 °C). After covering with breathable film, complete mycelial colonization occurred within 23 days, followed by 4–6 days of soil covering before mycelium emerged onto the soil surface (65–80 % RH). Primordia formed 12 days post-soil covering. During fruiting, maintaining 80–90 % RH supported normal development. The total growth cycle from inoculation to harvest was approximately 93 days, with two stable fruiting flushes per cultivation frame and 37 % biological efficiency under controlled conditions.

The cultivated fruiting bodies exhibited solitary growth, round pileus, strong aroma, and creamy white coloration. Morphological measurements showed 5 cm pileus diameters, short stipes (2.5 cm), and robust stipe thickness (>2.6 cm; Table 5).

## 4 Discussion

*Agaricus sinodeliciosus* is a newly identified species that was studied and named by Wang et al. in 2015 based on morphological and phylogenetic research [1]. Initially, it was believed to be primarily distributed in wetlands and grassy flats in northwest China [7]. The discovery of *A. sinodeliciosus* in Xinyuan County, Yili Kazakh Autonomous Prefecture, Xinjiang Uygur Autonomous Region, indicates an expansion of its ecological niche, allowing it to grow in sandy and saline-alkali soils in arid and semi-arid regions. The domestication and cultivation methods for *A. sinodeliciosus* are gradually being refined [9], 10].

In this study, the harvesting standard for *A. sinodeliciosus* fruiting bodies followed that of *Agaricus bisporus*, with a pileus around 5 cm in diameter, unopened, stem diameters close to 3 cm, and weights around 30 g. Although *A. sinodeliciosus* fruiting bodies can grow up to 1 kg, they are prone to opening and malformation, which affects quality. In this study, the pileus diameters of the harvested fruiting bodies were controlled at approximately 5 cm, rather than allowing unrestricted growth, resulting in a relatively low biological conversion efficiency. In future breeding and domestication efforts, a unified harvesting standard should be adopted to control fruiting body quality and increase the number of cultivation cycles and fruiting density to enhance yield.

*Agaricus sinodeliciosus* can grow under a wide range of C/N ratios, but its mycelium exhibits more vigorous and dense growth at a C/N ratio of 30:1. Therefore, the C/N ratio of the fermented substrate for frame cultivation should be adjusted to 30:1 to facilitate mycelial growth, primordium formation, and fruiting in *A. sinodeliciosus*. The optimal pH for *A. sinodeliciosus* is 7, but it can still grow at a pH of 9. Thus, the pH of the fermented substrate can be slightly alkaline, which not only supports mycelial growth but also inhibits the growth of contaminants. *A. sinodeliciosus* exhibits good tolerance to seasonal temperature fluctuations in different cultivation areas, with a wide coverage of the fruiting cycle. This broad adaptability to pH and temperature effectively reduces the strain’s dependence on special cultivation environments. It not only improves the cultivation error tolerance rate but also provides an important biological basis for the popularization and cultivation of this strain in regions with different climates and raw material conditions in China.

The preparation of spawn for *A. sinodeliciosus* often utilizes 94 % wheat grains [10]. In this study, the spawn formula was optimized. Through lignin-degrading enzyme activity tests, This study confirmed that *A. sinodeliciosus* exhibits good laccase and manganese peroxidase activities, which is consistent with the report of Hao et al. that the laccase from this species reaches maximum activity at 50 °C and pH 5.0 [16]. Therefore, in the spawn formula, we selected straw-based materials with high cellulose and low lignin content, such as corn straw, cottonseed hulls, and corn cobs. By incorporating straw-fermented substrate into the spawn formula, we reduced wheat grain usage by 50 % [10]. Moreover, the mycelial growth rate was faster, and the mycelium was denser compared with formulas using only cottonseed hulls and corn cobs. Further optimization of the spawn formula involved strictly controlling the fermented substrate’s C/N ratio at 30:1 and reducing the wheat content to 30 %. Under these conditions, the mycelium of *A. sinodeliciosus* was white and dense, grew rapidly, had an extremely low contamination rate, did not require post-ripening, and produced numerous and early primordia, facilitating soilless cultivation and fruiting.

The fermented substrate formulation for cultivating *A. sinodeliciosus* comprises 30 % corn straw, 23.5 % corn cob, 45 % cow dung, and 1.5 % lime for pH regulation, with a final carbon-to-nitrogen ratio of 30:1 and a moisture content of 65 %. *A. sinodeliciosus* has a broad carbon-to-nitrogen ratio tolerance and good ligninolytic enzyme activity, indicating its strong environmental adaptability. Corn straw, corn cob, and cow dung are all common agro-livestock production byproducts. This formulation achieves resource recovery through agricultural waste valorization, eliminates dependence on high-cost commercial substrates, significantly reduces raw material procurement costs, and combines technical feasibility with prominent economic advantages, thereby establishing a solid foundation for large-scale cultivation.

Li et al. reported that *A. sinodeliciosus* can produce fruiting bodies without casing soil using substrates such as rice straw, wheat straw, and reed [9]. In this study, the biological conversion efficiency and individual mushroom weight of fruiting bodies grown with casing soil were significantly higher than those grown without casing soil. Additionally, factors such as scattered light, oxygen levels, and moisture content during cultivation may influence pileus color. In this study, the cultivation of *A. sinodeliciosus* emulated the frame cultivation mode of *A. bisporus*, yielding two fruiting cycles. Regardless of whether casing soil was used, *A. sinodeliciosus* was able to produce two fruiting cycles. Cai et al. found that factors such as the water-holding capacity, porosity, and electrical conductivity of the casing soil influence mushroom yield [17]. In this experiment, peat soil was used as the casing material. Further research is needed to determine whether optimizing the proportion and thickness of peat soil mixed with garden soil in the casing material can enhance yield, considering that *A. sinodeliciosus* naturally grows in soil. The MW01 strain of *A. sinodeliciosus* can be further optimized through systematic breeding and refined cultivation management practices, such as double casing, to systematically optimize cultivation technological parameters and establish an efficient cultivation system. This approach aims to increase yield per unit area, enhance the number of fruiting cycles, and extend the overall cultivation period, thereby improving cultivation yield and economic benefits.

The cultivation of the *A. sinodeliciosus* enables effective absorption of locally available agricultural byproducts, facilitating on-site processing and disposal of agro-waste while creating additional income streams for farmers. Further exploration of post-cultivation spent mushroom substrate (SMS) utilization pathways – including composting for field application and secondary cultivation – can reduce waste discharge while converting SMS into organic fertilizer or secondary cultivation substrates. This establishes a complete eco-circular industrial chain from raw material supply to product reuse, demonstrating substantial potential for industrial-scale promotion and application. *A. sinodeliciosus* can enhance soil fertility, significantly influenced the distribution of soil bacteria and fungi, increasing the abundance of Streptomyces and reducing alpha diversity [18].

## 5 Conclusions

In this study, wild specimens of *A. sinodeliciosus* were collected from Yili region, Xinjiang Uygur Autonomous Region, and were identified through morphological and molecular biological methods. The research found that the optimal nitrogen source for *A. sinodeliciosus* is 10 g L^−1^ of cow manure, and the optimal C/N ratio is 30:1. The temperature range suitable for mycelial growth is 21–27 °C, and the optimal pH value is 7. *A. sinodeliciosus* has a strong ability to secrete manganese peroxidase and laccase. Based on its biological characteristics, effective formulations for mother culture media, stock culture media, and cultivation substrates were screened out, and an effective cultivation system was established. The gradual nationwide promotion of *A. sinodeliciosus* cultivation technology will facilitate its market-oriented development, thereby alleviating pressure on wild resource harvesting and achieving synergistic advancement in ecological conservation and industrial growth.
